# Factors associated with consulting a dietitian for diabetes management: a cross-sectional study

**DOI:** 10.1186/1472-6963-13-504

**Published:** 2013-12-05

**Authors:** Mohamad Alameddine, Lara Nasreddine, Nahla Hwalla, Yara Mourad, Hikma Shoaib, Dana Mousa, Farah Naja

**Affiliations:** 1 Faculty of Health Sciences, Department of Health Management and Policy, American University of Beirut, Riad El-Solh, Beirut 1107 2020, Lebanon; 2 Faculty of Agricultural and Food Sciences, Department of Nutrition and Food Sciences, American University of Beirut, Riad El-Solh, Beirut 1107 2020, Lebanon

**Keywords:** Planning and organizing care, Diabetes management, Human resources, Dietary counseling, Dietitian, Policy, Lebanon

## Abstract

**Background:**

Type 2 Diabetes (T2D) has reached epidemic levels in the Middle East region. Despite evidence that it improves health outcomes and saves health costs, dietary counseling for T2D remains grossly under-investigated in this region. The aim of this study was to assess the frequency and determinants of use of dietary counseling services by T2D patients in Lebanon and recommend corrective measures that may guide the planning, organization and delivery of care for chronic diseases in general and diabetes care in particular.

**Methods:**

A non-experimental cross-sectional design was utilized to survey outpatients with T2D in two major health centers in Lebanon. Patients diagnosed with T2D were invited to complete a questionnaire consisting of five sections: socio-demographic characteristics, disease attributes, patients’ perceptions regarding T2D management, practice of lifestyle modifications, and referral by a physician to a dietitian. The outcome of interest was the use of dietary counseling services by T2D patients at least once since their diagnosis. Descriptive statistics and logistic regression analyses were used to evaluate the frequency and determinants of dietary counseling services utilization.

**Results:**

A total of 332 T2D patients completed the questionnaire (response rate 94.6%). Although 75% of study participants believed that dietitians can assist them in changing their dietary habits, only 38% had consulted with a dietitian. Among study participants, only 34% were referred to a dietitian by their physician. The main determinants of the use of dietary counseling services were referral by a physician (OR: 112.25; 95% CI = 42.74-294.84), the presence of outpatient social or private health insurance (OR: 5.86; 95% CI = 2.40-14.25) and the belief that a dietitian can assist in changing dietary habits (OR: 3.74; 95% CI = 1.33-10.54).

**Conclusions:**

The findings of this study show suboptimal use of dietary counseling services by T2D patients in Lebanon. Key determinants were physicians’ referral, financial support for outpatient care, and patients’ belief in the usefulness of dietary counseling. Suggested interventions entail enhancing the planning and organization of care through inter-professional collaboration between physicians and dietitians; promoting public financing for high quality outpatient care that includes dietary counseling; and promoting the value of dietary counseling and improving the public image of dietitians.

## Background

Type 2 Diabetes (T2D) has reached epidemic levels in many countries of the Middle East [1], with prevalence rates ranging between 13.4% and 18.7% [2]. With a diabetes prevalence of 15.8% among adults older than 40 years, Lebanon is no exception [3]. This current prevalence of T2D poses a heavy burden on the healthcare system, due to the costs associated with the management of this disease and its complications [4,5]. Recently, multiple studies have highlighted the effectiveness and benefits of providing dietary counseling by a registered dietitian (RD) for T2D patients. These benefits include coordinating the lifestyle changes for patients [6-9], improving glycemic control (decreased mean HbA1c and reduced fasting plasma glucose) [9-13], reducing the need for oral medication or insulin [13], and enhancing cost-effectiveness through savings in drug therapy and medical services as well as through reduced expenditures for the management of diabetic complications [12,14,15].

Barriers to seeking dietary counseling services that have been reported in literature can be grouped within three main categories: personal/sociocultural barriers, professional barriers and financial barriers. From a socio-cultural perspective, it remains critical to improve the social image of the dietitian and reinforce the integral role that he/she plays in managing T2D and other diet-related chronic diseases. In general, patients seemed to expect dietary counseling from their physician rather than from dietitians [16], and when asked about their reasons for not visiting a dietitian, most patients reported apprehension and doubt towards the usefulness of dietary advice from dietitians [17].

On the professional level, family physicians seemed to perceive themselves as appropriately positioned to deliver health behavior counseling to their patients [18,19]. Although most physicians acknowledged the important role of diet in management of chronic diseases, only a few engaged dietitians in their patients’ care [16,20]. Physicians cited “limited access to dietitians” and “cost associated with dietary counseling” as main obstacles for referring to dietitians [21,22].

Refusal of insurance companies to reimburse dietary counseling continues to be a barrier for the utilization of these services. Evidence from the United States (US), has shown that the presence of health insurance increased utilization of outpatient physician services [23]. The research showed that cost-sharing, which requires patients to pay for a portion of healthcare costs not covered by health insurance, reduced the probability that they would use medical care [24]. Moreover, physicians expressed reluctance to refer patients to dietitians for health services not covered by public or private insurance plan [23,25]. Consequently and pointing to the scientific evidence supporting the efficacy of dietary counseling in diabetes management, the US Institute of Medicine recommended that dietary counseling provided by an RD be a covered Medicare benefit within a multidisciplinary approach to diabetes care [6]. This policy change has since led to a significant increase in the utilization of dietary counseling services [13,26,27].

Given the high prevalence rates of T2D in the Middle East region and the evident effectiveness of dietitians’ role in the management of the disease, it is crucial to integrate dietitians in the multidisciplinary team caring for T2D patients and increase the patients’ utilization of dietary counseling services. However, there is a paucity of research that has investigated dietary counseling with a dietitian for T2D management in the Middle East region in general and Lebanon in particular. The main objectives of this study were to assess the frequency of use of dietary counseling services by T2D patients in Lebanon and to determine the factors associated with consulting a dietitian among these patients. Concurrently, the study aimed at providing policymakers with insight into the enablers and barriers to optimizing the role of dietitians in the management of T2D patients.

## Methods

### Research design

A non-experimental cross-sectional study was designed to assess the utilization of dietary counseling services among T2D patients in Lebanon. The patients were recruited from out-patient clinics in two major medical centers in Beirut that have a high T2D patient load; a private not-for-profit teaching hospital and a large public hospital. Both medical centers are accredited by the Lebanese Ministry of Health. Ethics approval was obtained from the Institutional Review Board (IRB) for Social and Behavioral Sciences at the American University of Beirut.

### Study participants

Patients were eligible to participate in this study if they were: older than 18 years of age, had been diagnosed with T2D for at least one year prior to recruitment and have returned to one of the two hospitals, outpatient clinics to follow up with their treating physician. All the surveyed patients were visiting their physician for the management of their T2D. A sample size of 323 patients with T2D was required to obtain a 95% confidence interval of +/− 5% margin of error around a prevalence estimate of use of dietary counseling services in Lebanon of 30%. Consequently, and to account for drop outs, a total of 352 patients were invited to participate in the study.

Data collection took place between August and December 2010. In order to ensure a representative sample of patients from these centers, interviews were conducted on varying days and times of the week including week ends. A nurse and a graduate student in Nutritional Sciences carried out data collection. Both field workers had undergone extensive training in questionnaire administration and interviewing techniques and were clearly instructed and reminded to avoid asking any leading questions that could bias the patients’ answers in any particular direction. In addition, weekly meetings of the research team were held to ensure the inter-rater reliability and the standardization of data collection protocol. The nurses in charge at the clinic introduced the study to eligible patients. A member of the research team approached interested patients in the clinics’ waiting areas and explained to them the study objectives and protocol. In a one to one interview, participants completed the study questionnaire. Each participant was interviewed only once. Physicians involved in the patients’ treatment were not present during the interview. The participants were assured that any information they reveal would remain confidential and would be strictly used for research purposes. Patients were not paid to take part in the study and were informed that they were free to decline answering any questions they were not comfortable with. A written informed consent was obtained from each participating patient.

### Survey instrument

The survey instrument was divided into five sections: socio-demographic characteristics, disease attributes, patients’ perceptions regarding T2D management, practice of lifestyle modifications, and referral by a physician to a dietitian. Socio-demographic variables included age, gender, marital status, place of birth, educational status, employment status, crowding index (the ratio of the number of people over the number of rooms in the house), and presence of health insurance. The second section of the questionnaire included questions related to T2D such as age at diagnosis, duration of the disease, self-rated health status, presence of T2D complications, and family history of T2D. The third section tackled the patients’ perceptions of the role of diet/dietitians in the management of T2D. The fourth section of the questionnaire addressed the practice of lifestyle modifications pertaining to the management of T2D, which include adherence to a special diet or frequency of exercise. The last section focused mainly on referral to a dietitian and attendance of dietary counseling provided by a dietitian. The outcome of interest in this study- use of dietary counseling services by T2D patients at least once since diagnosis- was assessed by the following question: Have you ever consulted with a dietitian for the dietary management of your disease?

The content validity of the survey instrument was confirmed by a panel of experts consisting of one physician, one nutrition epidemiologist and one health policy expert. The original version of the questionnaire was written in English; yet since the questionnaire was planned to be administered to the patients in the Arabic language, it was translated to Arabic and back translated to English to ensure parallel-form reliability. The original and the back translated versions were reviewed for consistency in meaning by two bilingual experts. A pilot study was conducted with 15 selected T2D patients to ensure that the target population understood the questions, the wording was appropriate, and that the questions would yield the required data.

### Data analysis

Descriptive statistics, including frequencies and means, were reported for the socio-demographic characteristics, disease attributes, patients’ perceptions regarding T2D management and practice of lifestyle modifications for all study participants. The association of each of the characteristics of study participants with the use of dietetic services was assessed using a univariate logistic regression analysis. In order to evaluate the determinants of the use of dietary counseling services, a multivariate logistic regression model was used. Variables were put in the model in order of strength of their association with the outcome variable as per the univariate analysis and according to their reported importance in predicting the use of dietary counseling services in the literature. The effect of each variable on the model was assessed and the variable was kept in if it significantly contributed to a better fit of the model. The Hosmer and Lemeshow test was used to examine the goodness of fit of the final model. The Statistical Package for the Social Sciences software version 20.0 (SPSS Inc., Chicago, IL, USA) was used for all computations.

## Results

Out of 352 T2D patients invited to participate in this study, a total of 333 T2D patients completed the survey questionnaire (response rate 94.6%). Nineteen patients refused to participate, with the lack of time being the main reason behind refusal. One study participant had the question about the use of dietary counseling services missing and hence was only included in the overall descriptives and was excluded from the rest of the analysis. Among study participants (n = 332), 127 had ever consulted with a dietitian since diagnosis with T2D, resulting in a prevalence of use of dietary counseling services of 38.25% (95% CI = 33.04- 43.74). Of those patients who consulted with a dietitian (n = 127), only 33% (n = 42) did so more than one time.

### Descriptive analysis

The socio-demographic characteristics, disease attributes, patients’ perceptions regarding T2D management and practice of lifestyle modifications of the study participants are presented in Table 1. The average age of survey respondents was 60.18 ±11.90 years, with a comparable distribution by gender. The majority of participants were married (71.5%). Only 26.0% of subjects held a post-graduate degree, and the majority (66.4%) reported being unemployed (including housewives, retired and unemployed subjects). Regarding health insurance, over half of the study participants (56.3%) were under no coverage for outpatient visits.

**Table 1:** Comparison among T2D patients who consulted a dietitian and those who did not across a number of selected variables*

	**Independent variables**	**Overall n=333**^**+**^	**Consulted a dietitian n = 127**	**Did not consult a dietitian n = 205**	**OR (95% CI)**
**Socio-demographic characteristics**	Age (years)	60.18 ± 11.90	59.50 ± 11.91	60.63 ± 11.90	0.99(0.97-1.01)
	**Gender**				
	Male	184(55.3)	65(35.3)	119(64.7)	1
	Female	149(44.7)	62(41.9)	86(58.1)	1.32(0.85-2.06)
	**Marital status**				
	Not married	95(28.5)	38(40.4)	56(59.6)	1
	Married	238(71.5)	89(37.4)	149(62.6)	0.88(0.54-1.44)
	**Education**				
	Illiterate	39(11.8)	12(30.8)	27(69.2)	1
	Elementary School level	119(36.0)	40(33.9)	70(66.1)	1.15(0.53-2.52)
	High School level	87(26.3)	33(37.9)	54(62.1)	1.38(0.61-3.08)
	Diploma, graduate/postgraduate	86(26.0)	42(48.8)	44(51.2)	2.15(0.96-4.78)
	**Employment**				
	Unemployed	221(66.4)	87(39.5)	133(60.5)	1
	Employed	112(33.6)	40(35.7)	72(64.3)	0.85(0.53-1.36)
	**Crowding Index**				
	≤2 persons/room	251(75.8)	101(40.4)	149(59.6)	1
	>2persons/room	80(24.2)	24(30.0)	56(70.0)	0.63(0.37-1.09)
	**Place of birth**				
	Village/Town	142 (42.6)	54(38.0)	88(62.0)	1
	City	191(57.4)	73(38.4)	117(61.6)	1.02(0.65-1.59)
	**Presence of insurance for outpatient visits**				
	No	187(56.3)	56(30.1)	130(69.9)	1
	Yes	145(43.7)	71(49.0)	74(51.0)	*2.23(1.42-3.50)*
**Disease Attributes**	**Self-assessment of current health status**				
	Poor	66(19.8)	25(38.5)	40(61.5)	1
	Fair	157(47.1)	63(40.1)	94(59.9)	1.07(0.59-1.94)
	Good	110(33.0)	39(35.5)	71(64.5)	0.88(0.47-1.66)
	**Type of Clinic Attended**				
	Public	179(53.8)	53(29.8)	125(70.2)	1
	Private	154(46.2)	74(48.1)	80(51.9)	*2.18(1.39-3.42)*
	**Family history of diabetes**				
	No	151(45.3)	51(33.8)	100(66.2)	1
	Yes	182(54.7)	76(42.0)	105(58.0)	1.42(0.91-2.22)
	**Duration of T2D (years)**	11.08 ± 8.65	12.60 ± 9.23	10.11 ± 8.17	*1.03(1.01-1.06)*
	**Complications of Diabetes**				
	No	138(41.4)	46(33.3)	92(66.7)	1
	Yes	195(58.6)	81(41.8)	113(58.2)	1.43(0.91-2.26)
**Patients’ perceptions regarding T2D management**	**Importance of controlling blood glucose in managing T2D**				
	No/maybe it helps	43(12.9)	12(27.9)	31(72.1)	1
	It helps a lot	290(87.1)	115(39.8)	174(60.2)	1.71(0.84-3.46)
	**Importance of diet in controlling blood glucose levels**				
	No/maybe it helps	54(16.2)	15(27.8)	39(72.2)	1
	It helps a lot	279(83.8)	112(40.3)	166(59.7)	1.75(0.92-3.33)
	**Importance of exercise in controlling blood glucose levels**				
	No/maybe it helps	23(6.9)	9(39.1)	14(60.9)	1
	It helps a lot	310(93.1)	118(38.2)	191(61.8)	0.96(0.40-2.29)
	**A dietitian can assist in changing and regulating dietary habits**				
	No	77(25.2)	25(32.9)	51(67.1)	1
	Yes	229(74.8)	102(44.5)	127(55.5)	1.64(0.95-2.83)
**Practice of lifestyle modifications**	**Currently following a special diet**				
	No	167(50.2)	68(40.7)	99(59.3)	1
	Yes	166(49.8)	59(35.8)	106(64.2)	0.81(0.52-1.26)
	**Frequency of exercise per week**				
	<one time per week	195(58.6)	37(37.4)	62(62.6)	1
	≥one time per week	138(41.4)	90(38.6)	143(61.4)	0.86(0.55-1.36)

*Values in this table represent n and (%), except for age where means **±** (SD) are presented^+^. One study participant had the question about the use of dietary counseling services missing and hence was only included in the overall descriptives and was excluded from the rest of the analysis.

In reference to disease attributes, while 67% of study participants perceived their health status as either ‘fair’ or ‘poor’, only 33% reported having a ‘good’ health status. The sample population was evenly distributed between public (53.8%) and private (46.2%) clinics. The mean duration of T2D was 11 years, with more than half of the participants (58.6%) suffering from disease complications.

In terms of health beliefs, around 90% of the participants reported that they placed a high importance on proper blood sugar control in the management of T2D. Diet and exercise were perceived to play an important role in controlling blood sugar levels by 83.8% and 93.1% of respondents, respectively. Moreover, 75% of the participants in this study agreed that a dietitian is capable of assisting them in fostering dietary changes.

When asked about their current practice of lifestyle modifications, 50% of survey respondents indicated that they are not following any special diet and 58.6% reported exercising less than one time per week.

Table 1 also shows the results of the bivariate logistic regression describing the association between the variables previously described (as independent variables) and consulting with a dietitian (as a dependent variable). Among the patients’ socio-demographic characteristics, respondents who indicated having health insurance for outpatient visits had 2.23 times the odds of consulting with a dietitian compared to those who reported absence of it (OR: 2.23, 95% CI = 1.42-3.50). In relation to disease attributes, the type of clinic attended and the duration of T2D were both significantly associated with consulting with a dietitian. Participants attending private clinics were more likely to consult with a dietitian than those attending public clinics (OR: 2.18; 95% CI = 1.39-3.42). Furthermore, the odds of consulting with a dietitian were slightly higher with longer duration of the disease (OR: 1.03, 95% CI = 1.01-1.06).

**Figure 1:**
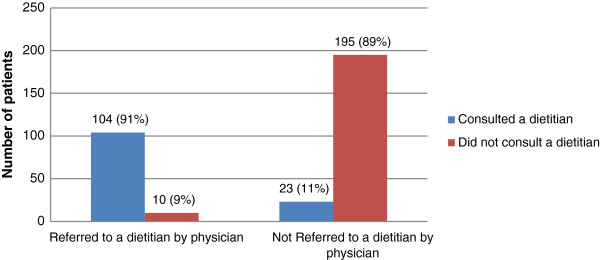
Use of dietary counseling among patients who were referred by physicians versus those who were not (n = 332).

Among study participants, only 34% were referred to a dietitian by their physician. Of those who were referred, the majority did consult with a dietitian (91%). On the other hand, out of all patients who were not referred by their physician, only 11% did consult with a dietitian (Figure 1).

### Multivariate analyses

Using multivariate logistic regression, two models were constructed to investigate determinants of consulting with a dietitian among T2D patients (Table 2). The first model included age, sex, duration of T2D, presence of diabetes complications, presence of health insurance, and the belief that a dietitian can assist in changing dietary habits. Model 2 included all the aforementioned variables in addition to ever being referred by a physician to a dietitian. Although the type of clinic attended (private or public) was significantly associated with the outcome variable, it was not included in neither of the two final models given its high association with the presence of health insurance for outpatient visits (r = 0.70). Nagelkerke’s Pseudo R^2^ was 0.20 and 0.72 for model 1 and model 2 respectively. Furthermore, Hosmer and Lemshow test indicated that both models had a good fit (Model 1: Chi square: 9.07, p = 0.0.38, Model 2: Chi square: 6.82, p = 0.556). Out of all the variables studied, referral by physicians emerged as a strong determinant, increasing the odds of consulting with a dietitian by more than a 100% (OR: 112.25; 95% CI = 42.74-294.84). Furthermore having health insurance coverage and believing that a dietitian can assist in changing dietary habits were both significantly associated with consulting a dietitian (OR: 5.86; 95% CI = 2.40-14.25 and OR: 3.74; 95% CI = 1.33-10.54, respectively).

**Table 2:** Determinants of consulting with a dietitian in the study sample using multivariate logistic regression models * (n = 332)

**Independent variables**	**Model 1 OR, 95% CI**	**Model 2 OR, 95% CI**
**Age** (years) (mean ± SD)	0.97, 0.95–1.00	0.98, 0.94–1.02
**Gender**		
Male	1	1
Female	1.27, 0.77–2.08	1.19, 0.54–2.59
**Duration of T2D** (mean ± SD)	*1.05, 1.02–1.08*	1.03, 0.98–1.08
**Presence of Complications of Diabetes**		
No	1	1
Yes	*2.02, 1.14–3.57*	1.33, 0.56–3.15
**Presence of insurance for outpatient visits**		
No	1	1
Yes	*4.20, 2.39–7.4*	*5.86, 2.40–14.25*
**Belief that a dietitian can assist in changing dietary habits**		
No/maybe it helps	1	1
It helps a lot	1.47, 0.82–2.63	*3.74, 1.33–10.54*
**Referral by the physician**		
No	Not included	1
Yes	Not included	*112.25, 42.73–294.84*

*Model 1 included the following variables: age, sex, duration of T2D, presence of diabetes complications, presence of health insurance, and the belief that a dietitian can assist in changing dietary habits. Model 2 included all the aforementioned variables in addition to ever being referred by a physician to a dietitian.

## Discussion

The study had a number of shortcomings that are worth mentioning. First, participants in this study were recruited from two major hospitals in Beirut (the capital of Lebanon). The socioeconomic background and the service structure in Beirut might limit the generalizability of our findings to rural and underserviced settings which have limited availability and access to dietary counseling services. Second, recruitment of patients from hospital settings might have increased the likelihood that these patients would have access to such services. Third, even with the explicit assurances that answers are confidential and anonymous, it cannot be ruled out that patients might have biased their answers in favor of physicians since interviews were conducted in the waiting room of the physicians’ clinics. Fourth, despite every effort by the research team to avoid asking the patients any leading question, the study results could potentially be biased by the fact that one of the interviewees has a background in nutrition. Yet, it is anticipated that the effect of the above-mentioned shortcomings will lead to an overestimation of the outcaome and that the utilization of the dietetic services might be in fact less than what has been reported in this study, which would further support the results and recommendations of this study. It is important to note that the cross-sectional design of the study does not support causal inferences but only associations.

Healthcare systems around the globe are striving for more effective and efficient delivery of quality health services [28]. Approaches to achieve this goal include strengthening primary health care, investing in health promotion and disease prevention initiatives, and proper management of chronic diseases - among others [29,30]. A multidisciplinary healthcare team is an essential element for the success of these approaches. While progress has been noted in developed and some developing countries in the effective utilization of the services of physicians and to a lesser extent nurses, the role of other health providers, especially dietitians, has not been properly integrated into the policies of health care systems [31,32]. This issue is even more pertinent to the Middle East region since most of its countries are suffering from an epidemic of diet-related diseases, including T2D [33-35].

The results of this study showed that, among Lebanese patients with T2D, only 38% visited a dietitian, of whom only 33% did so regularly. This finding is in agreement with previous reports from countries of the Middle East and North Africa (MENA) region. A study assessing the dietary practices among patients with diabetes in the United Arab Emirates revealed that 46% had never been seen by a dietitian since their diagnosis [33]. Furthermore, another study of Emirati and Omani adults with diabetes revealed that 25% of patients relied on informal networks such as friends and family for nutrition information. The authors highlighted the need to enhance the role of dietitians in providing diabetes education [36]. Moreover, results of a survey of private and public outpatient healthcare facilities in Qatar showed that the majority of diabetes programs were led by physicians while a few (17%) included dietitians' participation [37]. The underutilization of dietary counseling services clearly highlights a lost opportunity to benefit from the proven effectiveness of services of dietitians in the management of T2D [38]. These findings do not only cast doubt over the degree of integration of dietitians in the management of patients with T2D but also raise questions on the comprehensiveness and quality of outpatient care.

Moreover, our results indicated that consulting a dietitian was significantly associated with believing that a dietitian can assist in changing dietary habits. This finding is consistent with a large body of literature underscoring the significant impact patients’ psychological belief in the effectiveness of therapeutic regimens has on their compliance with these regimens [17,39]. Such an impact is particularly relevant should the compliance with the therapeutic regimen require long-term modifications to dietary and lifestyle habits, such is the case of T2D [17,40,41].

In the Lebanese context, the two main types of outpatient insurance coverage are social insurance for employed individuals (the National Social Security Fund) and private insurance [42,43]. Our analysis revealed a strong and significant association between seeking dietary services and the presence of social or private health insurance, whereby participants who possessed insurance had almost six times the odds of consulting a dietitian than those who did not. The lack of insurance coverage/ reimbursement has been previously documented to impede seeking dietary counseling on an outpatient basis and has been identified as a perceived barrier by patients with diabetes [16,23,44-46]. In the context of Lebanon, the higher odds of consulting a dietitian observed among those with health insurance, even if the insurance does not cover any dietary services, could be explained by the fact that those individuals are financially relieved from the bigger portion of healthcare cost associated with the disease treatment and would thus be more willing to pay out-of-pocket for the dietary consultation.

This study identified the referral by physician as an important factor that significantly influences the utilization of dietary counseling services by patients with T2D, whereby 91% of those who were referred by their physician did in fact visit the dietitian. Studies showed that interventions seeking to increase delivery of care by dietitians have only succeeded through solid partnerships with physicians who were committed to the referral system; when physicians’ commitment was lacking, utilization of dietary counseling services was often minimal [46,47]. The importance of strengthening physicians’ referral of patients with T2D to a dietitian is further underscored when physicians themselves reported that the amount of nutrition education received during their medical training is suboptimal and that they lack the sufficient knowledge of dietary counseling for T2D [19,21,38]. Furthermore, physicians rarely have time to allocate for one-to-one dietary counseling with their patients, and have often reported time as the predominant barrier to providing dietary counseling [16,23,38,48].

## Conclusions

Findings of this study highlighted the underutilization of dietary counseling services among patients with T2D in Lebanon, and identified referral by a physician, presence of insurance coverage for outpatient visits and patient’s belief in the usefulness of dietary counseling as determinants to seeking dietary counseling. Although some findings may be context-specific, we believe that the trends observed in this study may be extrapolated to other countries of the MENA region. National efforts should promote subsidized or affordable dietary counseling services, offered through the national network of primary healthcare centers to patients with T2D and those with other chronic diseases. The current lack of financial support for these services not only affects the outcomes of care but also casts doubt over the equity of access to dietary services in Lebanon.

Furthermore, the strong interrelationship between seeking dietary advice and being referred to a dietitian by a physician should encourage educators to emphasize the multidisciplinary approach to disease prevention and management and to ensure its integration within the curricula of medical studies.

Study findings also suggest a role for the Lebanese Academy for Nutrition and Dietetics [49], through its executive committee, in lobbying for a more prominent and integrated role for dietitians in primary healthcare. The Academy could also work on educating the public on the significant role that dietitians play in the management of chronic diseases; thus improving the public image of dietitians.

Dietitians represent an integral element within the health human resources matrix and are capable of fulfilling an active role in disease prevention and management, if provided the opportunity. Since diet is repeatedly reported as the most challenging aspect of diabetes care [44,50], why not delegate the task of delivering dietary and lifestyle education to healthcare personnel that are most qualified to do so? The findings of this study certainly plants the seeds for further investigations and for future actions by policymakers that would lay out a path for optimizing the role of dietitians in the management of T2D.

## Competing interests

The authors declare that they have no competing interests.

## Authors’ contributions

MA conceptualized the topic, led the write up of the proposal, contributed to data collection and analysis and prepared the first draft of the manuscript. LN helped with the conceptualization of the idea, contributed to data analysis and the write up of the manuscript. NH contributed to analysis and write up of the manuscript. YM contributed to literature review and synthesis and helped with the write up of the manuscript. HS contributed to the coding and cleanup of data collected, statistical analysis and write up. DM carried out data collection and contributed to data analysis and write up. FN co-led all aspects of the study and co-led the write up of the proposal and the write up of the manuscript. All authors read and approved the final manuscript.

## Pre-publication history

The pre-publication history for this paper can be accessed here:

http://www.biomedcentral.com/1472-6963/13/504/prepub
